# An Analytically and Diagnostically Sensitive RNA Extraction and RT-qPCR Protocol for Peripheral Blood Mononuclear Cells

**DOI:** 10.3389/fimmu.2020.00402

**Published:** 2020-03-20

**Authors:** Daniel J. Browne, Jamie L. Brady, Ashley J. Waardenberg, Claire Loiseau, Denise L. Doolan

**Affiliations:** ^1^Centre for Molecular Therapeutics, Australian Institute of Tropical Health & Medicine, James Cook University, Cairns, QLD, Australia; ^2^Centre for Tropical Bioinformatics and Molecular Biology, Australian Institute of Tropical Health & Medicine, James Cook University, Cairns, QLD, Australia

**Keywords:** PBMC, RNA, RT-qPCR, extraction, analytical-sensitivity, diagnostic-sensitivity

## Abstract

Reliable extraction and sensitive detection of RNA from human peripheral blood mononuclear cells (PBMCs) is critical for a broad spectrum of immunology research and clinical diagnostics. RNA analysis platforms are dependent upon high-quality and high-quantity RNA; however, sensitive detection of specific responses associated with high-quality RNA extractions from human samples with limited PBMCs can be challenging. Furthermore, the comparative sensitivity between RNA quantification and best-practice protein quantification is poorly defined. Therefore, we provide herein a critical evaluation of the wide variety of current generation of RNA-based kits for PBMCs, representative of several strategies designed to maximize sensitivity. We assess these kits with a reverse transcription quantitative PCR (RT-qPCR) assay optimized for both analytically and diagnostically sensitive cell-based RNA-based applications. Specifically, three RNA extraction kits, one post-extraction RNA purification/concentration kit, four SYBR master-mix kits, and four reverse transcription kits were tested. RNA extraction and RT-qPCR reaction efficiency were evaluated with commonly used reference and cytokine genes. Significant variation in RNA expression of reference genes was apparent, and absolute quantification based on cell number was established as an effective RT-qPCR normalization strategy. We defined an optimized RNA extraction and RT-qPCR protocol with an analytical sensitivity capable of single cell RNA detection. The diagnostic sensitivity of this assay was sufficient to show a CD8^+^ T cell peptide epitope hierarchy with as few as 1 × 10^4^ cells. Finally, we compared our optimized RNA extraction and RT-qPCR protocol with current best-practice immune assays and demonstrated that our assay is a sensitive alternative to protein-based assays for peptide-specific responses, especially with limited PBMCs number. This protocol with high analytical and diagnostic sensitivity has broad applicability for both primary research and clinical practice.

## Introduction

Reliable isolation of high quality and high quantity RNA from peripheral blood mononuclear cells (PBMCs) and other cells is critical for a broad range of basic, preclinical, and clinical applications (1, 2). RNA-based assays enable analysis of basal expression profiles and responses to antigen or mitogen stimulation (3, 4). Human PBMCs are a common source of RNA as collection of blood is less invasive and allows in-depth monitoring of many aspects of immunobiology (1, 5) including identification, classification and prognosis of cancers (6–11) monitoring inflammation (12, 13), and evaluating therapeutic efficacy (14–17).

A range of RNA-based platforms are now available, all dependent upon high quality and high quantity RNA (1). However, an important requirement for many applications is both excellent analytical sensitivity (i.e., smallest number of cells detectable) and diagnostic sensitivity (i.e., smallest detectable response to stimulation) (18). Protein-level immunoassays (e.g., flow cytometry, cytokine bead-based arrays, ELIspot) (19–22) are routinely used to detect PBMCs response to stimulation (23–25). Indeed, ELIspot has been used extensively as the “gold standard” immune assay given its sensitivity and has been optimized and validated as part of the global HIV/AIDS Comprehensive T Cell Vaccine Immune Monitoring Consortium (26–28). However, these protocols are limited by the relatively high number of cells required, especially when considering targets with low frequencies (24), when collection of large blood volumes is challenging (29, 30), or when there are many experimental variables [e.g., vaccine/peptide (14, 17, 31, 32) or epitope testing (33–35)]. Therefore, there is an unmet need for a robust RNA extraction and transcriptomic analysis protocol from limited input cell numbers (e.g., PBMCs) with high analytical and diagnostic sensitivity that meets or exceeds that of protein-level immuno-assays.

Reverse transcription quantitative PCR (RT-qPCR) remains the “Gold Standard” for assay of gene expression as an alternate readout to protein expression (36, 37). RT-qPCR is more sensitive than traditional RNA quantification technologies (i.e., Northern blotting, nuclease protection assays, *in-situ* hybridization, RNA microarrays etc.) (38–40). More recent technologies such as Sanger and next-generation sequencing (i.e., RNA-Seq, single cell RNA-seq, NanoString) and advanced PCR methods (i.e., digital PCR) are similarly sensitive (41, 42) but are relatively expensive or further require complex bioinformatical analysis (43, 44). In contrast, our optimized RT-qPCR assay is designed specifically for cheap, robust, reproducible and sensitive analysis of gene expression, is available to almost any laboratory, and serves as a sensitive and specific alternative to protein expression. Additionally, by focusing on a limited number of genes, RT-qPCR is ideal for validation of genes of interest identified from more untargeted methods such as RNAseq.

However, there is an unmet need for a robust RNA extraction and RT-qPCR protocol with excellent analytical and diagnostic sensitivity, ideally to the single cell level. An important consideration for such a protocol is that RT-qPCR normalization can be achieved by either absolute quantification of copies per reaction using a standard curve, or by semi-quantitative fold-change of relative expression normalized to reference genes (39, 45). However, *in vitro* stimulation has been shown to modulate the expression of many commonly used reference genes (46, 47), and key assumptions underlying semi-quantitative analysis require consistent reference gene expression across experimental conditions within and amongst cell populations. An alternative is absolute quantification normalized to cell number, which minimizes this potential analytical bias (48–50).

To address this need, we developed a highly sensitive RNA extraction and RT-qPCR quantification strategy for analysis of gene expression from human PBMCs. We compared the efficiency of the latest generation of SYBR master-mixes and RNA extraction and reverse transcription kits, taking into consideration both total RNA yield and RNA concentration. We determined that ssoAdvanced™ Universal SYBR® Green Master-Mix provided optimal reaction efficiency, whilst SuperScript™ IV Reverse Transcriptase had the highest cDNA yields. We demonstrated significantly increased PBMC RNA recovery following extraction with the magnetic bead-based MagMAX™ *mirVana*™ kit, with no further enhancement of analytical sensitivity by including an additional step of RNA concentration. When testing the analytical sensitivity of our optimized protocol, we could detect RNA to the single cell level of highly expressed genes. Furthermore, by evaluating a hierarchy of CD8^+^ T cell epitope responses, we demonstrated diagnostic sensitivity with as few as 1 × 10^4^ PBMCs. This optimized RNA extraction and RT-qPCR protocol, with high analytical and diagnostic sensitivity, provides a robust alternative to protein-based immune assays.

## Materials and Equipment

### PBMC Stimulatory Reagents

- Phorbol 12-Myristate 13-Acetate (PMA), (Sigma-Aldrich)
- Ionomycin (Iono), (Sigma-Aldrich)
- Phytohemagglutinin-L (PHA), (Sigma-Aldrich)
- Human Cytomegalovirus, Epstein Barr Virus and Influenza virus Synthetic peptides (Table 1).

**Table 1:** Synthetic peptides.

**Code**	**Amino acid sequence**	**HLA resriction**	**Species**
*VTE*	VTEHDTLLY	A1	Cytomegalovirus
*GIL*	GILGFVFTL	A2	Influenza
*RPH*	RPHERNGFTVL	B7	Cytomegalovirus
*FLR*	GILGFVFTL	B8	Epstein Barr Virus

*Characteristics of synthetic peptides representing defined CD8^+^ T cell peptide epitopes derived from CMV, EBV and influenza virus restricted by the MHC-class I molecules HLA-A1, -A2, -B7, and -B8*.

### SYBR Mastermix Kits:

- ssoAdvanced™ Universal SYBR® Green Master-Mix (Bio-Rad)
- QuantiNova SYBR® Green PCR Kit (QIAGEN).
- PowerUp SYBR® Green Master-Mix (Applied Biosystems)
- RT^2^ SYBR® Green qPCR Master-Mix (QIAGEN).

### RNA Extraction Kits

- RNeasy® Mini Kit (QIAGEN)
- RNeasy® MiniElute Cleanup Kit (QIAGEN)
- RNeasy® Micro Kit (QIAGEN)
- MagMAX™ *mirVana*™ Total RNA Isolation Kit (Applied Biosystems).

### RNA to cDNA Synthesis Kits

- SuperScript™ III First-Strand Synthesis System (ThermoFisher)
- SuperScript™ IV First-Strand Synthesis System (ThermoFisher)
- iScript™ Advanced cDNA Synthesis Kit (Bio-Rad)
- High-Capacity RNA-cDNA Kit™ (Applied Biosystems).

### Quantitative PCR Primers

- PrimerBank™ primers (Table 2).

**Table 2:** Primer list.

**Transcript**	**GenBank accession number**	**PrimerBank™ ID**	**Forward sequence (5′-3′)**	**Reverse sequence (5′-3′)**	**Amplicon size (bp)**
**Reference genes**					
*RPLA13a*	NM_012423	14591905c2	GCCCTACGACAAGAAAAAGCG	TACTTCCAGCCAACCTCGTGA	117
*SDHA*	NM_004168	156416002c3	TGGCATTTCTACGACACCGTG	GCCTGCTCCGTCATGTAGTG	77
*TBP*	NM_003194	285026518c2	CCCGAAACGCCGAATATAATCC	AATCAGTGCCGTGGTTCGTG	80
**Cytokine gene**					
*IFN-γ*	NM_000619.2	56786137c1	TCGGTAACTGACTTGAATGTCCA	TCGCTTCCCTGTTTTAGCTGC	93

*Characteristics of primers acquired from PrimerBank™ database*.

### Antibodies

- anti-human IFN-γ monoclonal antibody (mAb) (Clone 1-D1K, MABTECH)
- anti-human IFN-γ biotinylated mAb (Clone 7-B6-1, MABTECH)
- anti-human IFN-γ-FITC mAb (Clone 4S.B3, BD Biosciences).

### Equipment

- QuantStudio 3 Real-Time PCR system (Applied Biosystems)
- NanoDrop 2000 Spectrophotometer (ThermoFisher)
- 2100 Bioanalyzer (Agilent Technologies)
- AID ELIspot reader system (Autoimmun Diagnostika GmbH, Germany)
- LSRFortessa X-20 (BD Biosciences).

### Software

- QuantStudio Design and Analysis Software (v1.4.3, Applied Biosystems)
- ProcartaPlex Analyst Software (v1.0, ThermoFisher)
- FlowJo Software (v10.4, BD Biosciences)
- GraphPad Prism (v7, GraphPad).

## Methods

### Samples

#### PBMCs

Blood was collected from healthy donors or buffy coats (*n* = 12) provided by the Australian Red Cross Blood Service, under a protocol approved by the James Cook University Human Research Ethics Committee (#H6702). PBMCs were isolated by density gradient centrifugation and cryopreserved in FBS 10% DMSO. Prior to use, samples were thawed rapidly at 37°C, treated with DNAase I (1 μg/mL; StemCell), and rested for 18 h at 2 × 10^6^ cells/mL in media (RPMI-1640, 10% FBS, 100 U/mL penicillin/streptomycin) at 37°C and 5% CO_2_. Viable PBMCs were counted prior to downstream analysis.

#### HLA Typing

Genomic DNA was isolated from PBMCs using the QIAamp DNA Mini Kit (QIAGEN) according to manufacturer's instructions. High-resolution class I and class II HLA typing was performed by the Australian Red Cross Transplant and Immunological Services (Melbourne, Australia) using the MIA FORA NGS FLEX HLA typing kit (Immunocor) and Illumina MiSeq and MiniSeq platforms.

#### Cell Stimulation

PBMCs were resuspended in RPMI-1640 supplemented with 10% human serum, 100 U/mL penicillin/streptomycin, 2 mM glutaMAX (ThermoFisher Scientific), 10 mM HEPES (ThermoFisher Scientific), and 5 × 10^−5^ M β-Mercaptoethanol (Sigma-Aldrich) (complete media). Synthetic peptides (10 μg/mL) representing defined CD8^+^ T cell epitopes from human Cytomegalovirus, Epstein Barr Virus or Influenza virus (Table 1) were tested alongside PMA/Iono (50 ng/mL PMA, 1,000 ng/mL Iono) and PHA (PHA; 1 μg/mL) mitogen controls as well as media-only negative control. PBMCs were stimulated for 6, 12, 16, 24, or 48 h at 2 × 10^6^ cells/mL in 200 μL in 96-well U-bottom plates (qPCR, ELIspot and multiplexed bead array) or at 1 × 10^6^ cells/mL in 3 mL in 12-well flat-bottom plates (flow cytometry).

### Quantitative PCR

#### Assay Setup

qPCR was conducted using the QuantStudio 3 Real-Time PCR system running QuantStudio Design and Analysis Software (v1.4.3, Applied Biosystems). A standard curve, combined calibration sample, and no template negative controls were included on each plate. All samples were run in technical triplicate in accordance with MIQE guidelines (45). cDNA synthesis was conducted on a SimpliAmp™ thermocycler (ThermoFisher Scientific). Unless specifically noted, all reaction conditions and protocols were performed as recommended by the manufacturer. Copies/reaction were determined by absolute quantification.

#### SYBR Master-Mix Testing: Amplicon Standard

Four SYBR master-mix kits were evaluated: ssoAdvanced™ Universal SYBR® Green Master-Mix (Bio-Rad), QuantiNova SYBR® Green PCR Kit (QIAGEN), PowerUp SYBR® Green Master-Mix (Applied Biosystems) and RT^2^ SYBR® Green qPCR Master-Mix (QIAGEN). *RPL13a, SDHA, TBP*, and *IFN-*γ primer (Table 2) amplicons were purified by Wizard SV Gel & PCR CleanUp System (Promega) and quantified by NanoDrop 2000 Spectrophotometer (ThermoFisher). Master-mix reaction efficiency was calculated by amplification of amplicons titrated from 10^7^ to 10^1^ copies/reaction, with primers at 250, 500, or 750 nM (45).

#### SYBR Master-Mix Testing: cDNA Standard

The four SYBR master-mix kits were further evaluated with efficiency titrations of cDNA standards. Briefly, RNA was extracted from 1 × 10^6^ unstimulated PBMCs using the RNeasy® Mini Kit (QIAGEN). Seven microliters of extracted RNA was converted to cDNA using the SuperScript™ III First-Strand Synthesis System kit (Invitrogen). Master-mix reaction efficiency was calculated from log_10_ diluted cDNA (10^4^-10^1^ cells/reaction) with *RPL13a, SDHA, TBP*, and *IFN-*γ primers at 500 nM.

#### Reference Gene Stability Testing

1 × 10^6^ PBMCs were stimulated for 6, 12, 16, 24, or 48 h with or without PMA/Iono as described above. RNA was extracted with RNeasy® Mini Kit (QIAGEN). Seven microliters of extracted RNA was reverse transcribed with SuperScript™ III First-Strand Synthesis System kit (Invitrogen). qPCR was run with ssoAdvanced™ master-mix, *RPL13a, SDHA, TBP* and *IFN-*γ primers at 500 nM and samples at 10^2^ cells/reaction.

#### Evaluation of RNA Extraction Kits

To evaluate RNA yield and quality, three RNA extraction kits: RNeasy® Mini Kit (QIAGEN), RNeasy® Micro Kit (QIAGEN), and MagMAX™ *mirVana*™ Total RNA Isolation Kit (Applied Biosystems); and one post-extraction RNA purification and concentration kit, RNeasy® MiniElute Cleanup Kit (QIAGEN), were evaluated. All extractions included genomic DNA removal. RNA was extracted from 1 × 10^6^ PBMCs incubated for 6 h with or without PMA/Iono, with the exception of the RNeasy® Micro Kit where 0.5 × 10^6^ PBMCs was used (per manufacturer's recommendation). To evaluate concentration, kit eluates were concentrated using the RNeasy® MiniElute. All elutions were performed in the smallest recommended volume. The yield of extracted RNA was quantified using a NanoDrop 2000 Spectrophotometer (ThermoFisher). RNA yield (i.e., total RNA extracted), calculated RNA quality was assessed by RNA integrity number (RIN) by the Australian Genome Research Facility (Brisbane, Australia) using a 2100 Bioanalyzer (Agilent Technologies). Subsequently, 7 μL of RNA was converted to cDNA using the SuperScript™ III First-Strand Synthesis System kit (Invitrogen) alongside negative reverse transcriptase controls. qPCR was run with ssoAdvanced™ master-mix, *RPL13a, SDHA, TBP*, and *IFN-*γ primers at 500nM and sampled at 10^2^ cells/reaction.

#### Evaluation of Reverse Transcription Kits

Four reverse transcription kits were evaluated: SuperScript™ III First-Strand Synthesis System (ThermoFisher Scientific), SuperScript™ IV First-Strand Synthesis System (ThermoFisher), iScript™ Advanced cDNA Synthesis Kit (Bio-Rad) or High-Capacity RNA-cDNA Kit™ (Applied Biosystems). Kits were evaluated using RNA extracted using the MagMAX™ *mirVana*™ Total RNA Isolation Kit (MagMAX) with or without the RNeasy® MiniElute Cleanup Kit. Briefly, 1 × 10^6^ PBMCs were incubated for 6 h with or without PMA/Iono as described above. Seven microliter of RNA extracted by MagMAX was used for each cDNA synthesis kit. Alternatively, the maximum recommended input of RNA extracted by MagMAX in association with the RNeasy® MiniElute Cleanup Kit was used (i.e., Superscript™ III 7 μL, Superscript™ IV 10 μL, iScript™ 14 μL, and High-Capacity 9 μL). qPCR was run with ssoAdvanced™ master-mix, *RPL13a, SDHA, TBP*, and *IFN-*γ primers at 500 nM and sample diluted to 10^2^ cells/reaction; except when considering concentration, when the samples were run undiluted.

#### Analytical and Diagnostic Sensitivity

For determination of analytical sensitivity, RNA was extracted from a log_10_ serial dilution of unstimulated PBMCs (10^6^-10^0^ cells/extraction), using the MagMAX kit with or without the RNeasy® MiniElute Cleanup Kit. A media-only extraction control was processed in parallel. For determination of diagnostic sensitivity, RNA was extracted using the MagMAX kit from titrated PBMCs (4 × 10^5^, 1 × 10^5^, 2.5 × 10^4^, and 1 × 10^4^) incubated for 6 h with or without PMA/Iono or HLA-matched peptide. For sensitivity evaluations, 10 μL of RNA was converted to cDNA using the SuperScript™ IV First-Strand Synthesis System (Invitrogen). qPCR used undiluted sample with ssoAdvanced™ master-mix, *RPL13a, SDHA, TBP*, and *IFN-*γ primers at 500 nM.

### Protein Quantification Assays

#### Enzyme-Linked Immunospot (ELIspot) Assay

IFN-γ ELIspot assays were performed as previously described (51, 52). Briefly, 4 × 10^5^ PBMCs were plated in triplicate onto 96-well multi-screen filtration plates (#MAIP S45-10, Merck) pre-coated with anti-human IFN-γ monoclonal antibody (mAb) (Clone 1-D1K, MABTECH) and stimulated for 24 h with or without peptide, PMA/Iono, PHA or media. After washing, IFN-γ secreting cells were stained with 1 μg/mL of anti-human IFN-γ biotinylated mAb (Clone 7-B6-1, MABTECH) followed by streptavidin alkaline phosphatase (MABTECH). The assay was developed using the AP Conjugate Substrate Kit (BioRad). IFN-γ-spot-forming cells were counted using AID ELIspot reader system (Autoimmun Diagnostika GmbH, Germany).

#### Multiplex Cytokine Bead Array

Supernatant was collected from 4 × 10^5^ PBMCs incubated for 6 h with or without peptide, PMA/Iono or media. Fifty microliter supernatant was analyzed using the ProcartaPlex Immunoassay (ThermoFisher) per manufacturer's protocol. Cytokine concentration was calculated from a standard curve using the ProcartaPlex Analyst 1.0 Software (ThermoFisher).

#### Flow Cytometry

3 × 10^6^ PBMCs were incubated for 6 h with or without peptide, PMA/Iono or media. 5 μg/mL brefeldin A (BD Biosciences) was added after 1 h. Cells were then stained with Fixable Viability Stain 510 (BD Bioscience) and permeabilizated with the Cytofix/Cytoperm kit (BD Biosciences) before staining with anti-human IFN-γ-FITC (Clone 4S.B3, BD Biosciences) mAb. Data were acquired on a LSRFortessa X-20 driven by FACSDiva software (BD Biosciences) and analyzed using FlowJo software (version 10.4).

### Data Analysis

RT-qPCR, Bioanalyzer and NanoDrop data were analyzed using a repeated-measures two-way ANOVA with Bonferroni-corrected multiple comparisons test comparing test to control mean. Correlation between RT-qPCR and protein quantification was test with linear regression analysis. Analysis was conducted using GraphPad Prism version 7.0 (GraphPad). In all cases, *P* < 0.05 were considered significant.

## Results

### ssoAdvanced™ Universal SYBR® Green Master-Mix Provided the Highest Reaction Efficiency

**Figure 1:**
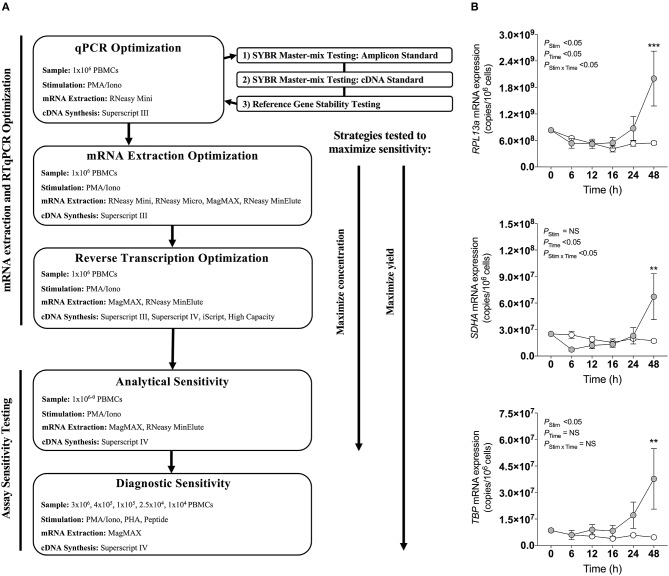
qPCR optimization. **(A)** Experimental workflow for qPCR optimization. **(B)** Effect of stimulation on mRNA expression of reference genes *RPL13a, SDHA*, and *TBP*. 1 × 10^6^ PBMCs paired samples were cultured with complete media (white), or stimulated with PMA/Iono control (gray) for 0, 6, 12, 16, 24, or 48 h. RNA was extracted using the RNeasy® Mini (Mini) Kit, and reverse transcribed with Superscript™ III (Invitrogen). RNA expression was determined by absolute quantitative RT-qPCR wherein number of gene copies per reaction was quantified by standard curve and normalized to cell number. Data were compared with a two-way ANOVA with *post-hoc* Bonferroni's multiple-comparisons test (**P ≤ 0.01; ***P ≤ 0.001). Biological replicate (*n* = 3) single RNA extractions with single reverse transcription reactions per extraction were performed. Sample mean calculated from technical triplicate qPCR. Biological mean ± biological SEM are shown.

Four master-mixes—ssoAdvanced™ Universal SYBR® Green Master-Mix (Bio-Rad), QuantiNova SYBR® Green PCR Kit (QIAGEN), PowerUp SYBR® Green Master-Mix (Applied Biosystems) and RT^2^ SYBR® Green qPCR Master-Mix (QIAGEN)—were evaluated using two methods of preparing reference standards: (i) standards derived from log_10_ diluted amplicon; and (ii) standards generated from log_10_ diluted cDNA (Figure 1A). Reaction efficiency was quantified using four primer sets: three sets targeted reference genes known to have high (60S ribosomal protein L13a; *RPL13a*), moderate (Succinate dehydrogenase complex, subunit A; *SDHA*) and low (TATA-binding protein; *TBP)* expression; and one set targeted a cytokine gene (interferon gamma; *IFN-*γ) (47). When considering an acceptable reaction efficiency range (90–110%), 35.4% of the amplicon-derived standards (Table 3) and 43.8% of the diluted cDNA standards (Table 4) failed. Primer concentration did not significantly affect mean deviation from 100% reaction efficiency. Strikingly, when comparing SYBR master-mix kits with cDNA standards, the use of ssoAdvanced™ had 0% failure (efficiency*: RPL13a* 93.7%, *SDHA* 98.3%, *TBP* 95.9%, and *IFN-*γ 96.1%), the largest dynamic range (10^0−4^ copies/reaction), and the lowest mean deviation from 100% (Table 4). The coefficient of determination of the standards (*R*^2^ ≥ 0.97) were consistent for all primers tested, at all concentrations. Together, these data identify ssoAdvanced™ master-mix as providing the highest reaction efficiency for qPCR from PBMC cDNA.

**Table 3:** Evaluation of commercial qPCR master-mixes by amplicon derived standards.

		**QIAGEN** ***R***^****2****^ **SYBR master-mix**			**QIAGEN QuantiNOVA SYBR Master-mix**			**Bio-rad ssoadvanced SYBR Master-mix**			**Applied biosystems PowerUp SYBR master-mix**			
**[Primer]**	**Transcript**	**E′ (%)**	**LOD (copies/reaction)**	***R*^**2**^**	**E′ (%)**	**LOD (copies/reaction)**	***R*^**2**^**	**E′ (%)**	**LOD (copies/reaction)**	***R*^**2**^**	**E′ (%)**	**LOD (copies/reaction)**	***R*^**2**^**	**Primer failure rate (%)**
250 nM	*RPLA13a*	92.2	10^1^	0.99	94.9	10^2^	0.99	89.1	10^1^	0.99	92.0	10^1^	0.99	31.3
	*SDHA*	107.2	10^1^	0.99	102.1	10^1^	0.99	94.3	10^1^	0.99	94.5	10^1^	0.99	
	*TBP*	86.1	10^1^	0.99	94.7	10^1^	0.99	90.0	10^1^	0.99	89.7	10^1^	0.99	
	*IFN-γ*	93.6	10^1^	0.99	85.8	10^1^	0.98	83.9	10^2^	0.99	99.7	10^1^	0.98	
250 nM failure rate		25.0%			25.0%			50.0%			25.0%		
500 nM	*RPLA13a*	88.8	10^1^	0.99	96.9	10^1^	0.99	86.0	10^2^	0.99	92.7	10^1^	0.99	37.5
	*SDHA*	93.8	10^1^	0.99	93.5	10^2^	0.99	92.4	10^1^	0.99	90.8	10^1^	0.99	
	*TBP*	88.6	10^1^	0.99	82.6	10^2^	0.99	89.2	10^1^	0.99	98.4	10^1^	0.99	
	*IFN-γ*	93.5	10^1^	0.99	87.1	10^2^	0.99	92.4	10^1^	0.99	95.2	10^1^	0.99	
500 nM failure rate		50%			50%			50%			0%		
750 nM	*RPLA13a*	98.2	10^1^	0.99	87.7	10^2^	0.99	96.8	10^1^	0.99	94.0	10^1^	0.99	37.5
	*SDHA*	98.0	10^1^	0.99	104.2	10^1^	0.99	102.1	10^1^	0.99	92.3	10^2^	0.97	
	*TBP*	86.7	10^2^	0.99	89.2	10^1^	0.99	87.2	10^1^	0.99	85.5	10^2^	0.99	
	*IFN-γ*	94.4	10^1^	0.99	84.0	10^1^	0.99	92.3	10^1^	0.99	96.3	10^1^	0.99	
750 nM failure rate		25.0%			75.0%			25.0%			25.0%			
Master-mix failure rate		33.3%			50.0%			41.7%			16.7%			
Overall failure rate		35.4%												

*Commerical qPCR master-mixes were evaluated in combination with three primer concentrations (250, 500, and 750 nM) with logarithmically-diluted amplicon derived standards (10^6^ to 10^1^ copies amplicon per reaction). Assay performance i.e., reaction efficiency (E′), limit of detection (LOD) and standard coefficient of determination (R^2^) determined as per MIQE guidelines. Amplicons were purified once and measured in qPCR technical triplicate. E′, Dynamic Range and R^2^ were calculated from the mean of the qPCR technical triplicates. Failure rate calculated as percentage outside accceptable E′ (90–110%)*.

**Table 4:** Evaluation of commercial qPCR master-mixes by cDNA derived standards.

	**QIAGEN** ***R***^****2****^ **SYBR master-mix**			**QIAGEN QuantiNOVA SYBR master-mix**			**Bio-Rad ssoAdvanced SYBR master-mix**			**Applied biosystems PowerUp SYBR master-mix**		
**Transcript**	**E′ (%)**	**Dynamic range (cells/reaction)**	***R*^**2**^**	**E′ (%)**	**Dynamic range (cells/reaction)**	***R*^**2**^**	**E′ (%)**	**Dynamic range (cells/reaction)**	***R*^**2**^**	**E′ (%)**	**Dynamic range (cells/reaction)**	***R*^**2**^**
*RPL13a*	95.2	10^0^–10^3^	0.99	78.9	10^0^–10^3^	0.99	93.7	10^0^–10^4^	0.99	104.9	10^0^–10^3^	0.99
*SDHA*	79.9	10^0^–10^3^	0.99	85.7	10^0^–10^3^	0.99	98.3	10^0^–10^4^	0.99	121.5	10^0^–10^3^	0.99
*TBP*	134.7	10^2^–10^4^	0.99	99.1	10^2^–10^4^	0.99	95.9	10^1^–10^4^	0.99	109.7	10^2^–10^4^	0.99
*IFN–γ*	88.3	10^1^–10^3^	0.99	91.2	10^1^–10^3^	0.99	96.1	10^1^–10^4^	0.99	121.9	10^0^–10^3^	0.99
Master-mix failure rate	75.0%			50.0%			0.0%			50.0%		
Overall failure rate	43.8%											

*Commerical qPCR master-mixes were evaluated with logarithmically diluted PBMC cDNA (10^4^-10^1^ cell cDNA equivalent per reaction). Assay performance, i.e., reaction efficiency (E′), limit of detection (LOD) and standard coefficient of determination (R^2^) determined as per MIQE guidelines. Biological replicates (n = 3) were extracted with single reverse transcription reactions per extraction, and measured in qPCR technical triplicate. RNA extractions were repeated. E′, Dynamic Range and R^2^ calculated from a representative technical extraction, calculated from the mean of a qPCR technical replicate. Failure rate calculated as percentage outside accceptable E′ (90–110%)*.

### Mitogen Stimulation Induced Changes in RPL13a, SDHA, and TBP Gene Expression

The expression stability of three commonly used reference genes (47, 53, 54), *RPL13a, SDHA*, and *TBP*, previously reported as stable in PBMCs following stimulation (47), were evaluated by RT-qPCR within PBMCs stimulated with PMA/Iono for 6, 12, 18, 24, or 48 h. Expression of all three genes changed over time with cell culture, and significantly increased at 48 h post-stimulation as compared to baseline (*P* < 0.001*, P* < 0.01, and *P* < 0.01, respectively; Figure 1B). These data establish that the expression of common reference genes is significantly affected by stimulation, emphasizing the importance of absolute quantification normalized to cell numbers, rather than relative quantification.

### Magnetic Bead-Based Extraction Significantly Increased RNA Yield and Concentration

**Figure 2:**
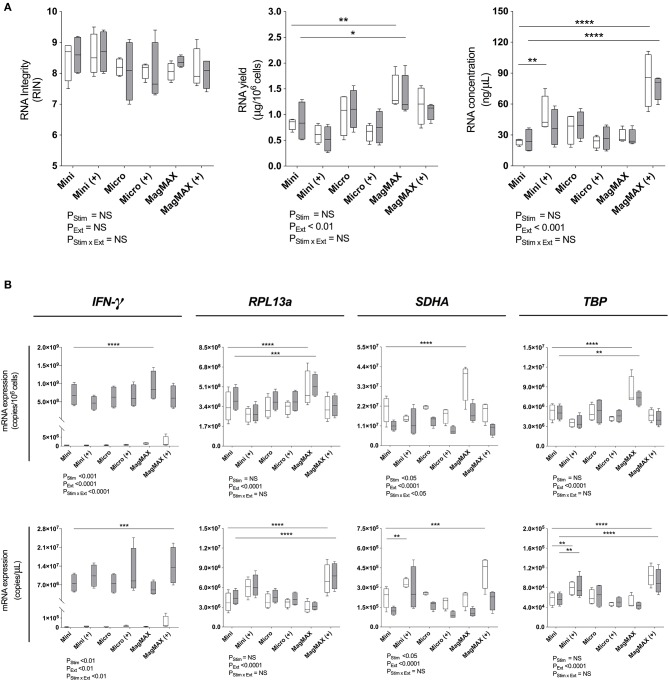
RNA extraction evaluation. **(A)** Bioanalyser analysis of RNA Integritry Number (RIN), and nanospectrophotometer analysis of yield and concentration were obtained using three commercially-available extraction kits with (+) or without post-extraction RNA purification and concentration. **(B)** RT-qPCR analysis of *IFN-*γ, *RPL13a, SDHA*, and *TBP* expression normalized to cell number (copies/10^6^ cells) or cDNA concentration (copies/μL). 1 × 10^6^ PBMCs paired samples were cultured with complete media (white) or PMA/Iono (gray) for 6 h. RNA was extracted using the RNeasy® Mini (Mini) Kit, RNeasy® Micro (Micro) Kit (both QIAGEN), or MagMAX™ *mirVana*™ (MagMAX) Total RNA Isolation Kit (Applied Biosystems), with concentration step performed using the RNeasy® MiniElute Cleanup Kit (QIAGEN). All samples were reverse transcribed with Superscript™ III (Invitrogen). Data were analyzed using a two-way ANOVA with *post-hoc* Bonferroni's multiple-comparisons test (*P ≤ 0.05; **P ≤ 0.01; ***P ≤ 0.001; ****P ≤ 0.0001). Biological replicate (*n* = 4), triplicate RNA extractions, with single reverse transcription reactions per extraction were performed. Sample mean calculated from the mean of the technical RNA extractions which were in turn calculated from the mean of the technical triplicate qPCR reactions. Biological mean ± biological SEM are shown.

Next, RNeasy® Mini and Micro silica columns (both QIAGEN) and MagMAX™ *mirVana*™ (MagMAX) Total RNA Isolation (Applied Biosystems) kits were tested for 1) RNA yield and 2) concentration with or without a post-extraction RNA concentration step using the RNeasy® MiniElute Cleanup Kit (QIAGEN). In each case, PBMCs were incubated with or without PMA/Iono for 6 h. RIN assessment demonstrated that RNA integrity was high (>7) and consistent across all kits (Figure 2A, left panel). RNA yield was significantly increased using MagMAX as compared to the RNeasy® Mini silica column extraction kit, for both stimulated and unstimulated PBMCs (mean yield (μg/10^6^ cells): 0.87 vs. 1.36, *P* < 0.05 and 0.82 vs. 1.42, *P* < 0.01, respectively) (Figure 2A, middle panel). Moreover, the concentration of RNA extracted from both stimulated and unstimulated PBMCs significantly increased with the MagMAX-RNeasy® MiniElute combination (mean RNA concentration (ng/μL): 23.5 vs. 83.9, *P* < 0.0001 and 24.8 vs. 76.6, *P* < 0.0001, respectively) (Figure 2A, right panel). The RNeasy® Micro extraction kit had no impact on RIN, RNA yield or concentration.

### Magnetic Bead-Based Extraction Significantly Increased RT-qPCR Gene Expression Signal Measurements

RNA extraction kit eluates were subsequently reverse transcribed using Superscript™ III (ThermoFisher) and *RPL13a, SDHA, TBP* as well as *IFN-*γ quantified by RT-qPCR. Data were normalized to either RNA yield (copies/10^6^ cells) or concentration (copies/μL). When considering both yield and concentration, RT-qPCR signal was significantly affected by extraction technique (*P*_*Ext*_ < *0.01* for all tested genes; Figure 2B). Consistent with our findings that *SDHA* expression was reduced following 6 h of exposure to PMA/Iono (Figure 1B), stimulation significantly reduced *SDHA* expression (*P* < *0.05;* Figure 2B). Similarly, consistent with our findings that RNA yield was optimal with MagMAX (Figure 2A), we found significantly increased gene expression using MagMAX when compared to RNeasy® Mini, for both unstimulated (mean copies/10^6^ cells: 3.4 × 10^8^ vs. 4.9 × 10^8^, *P* < 0.0001 *RPL3a;* 2.1 × 10^7^ vs. 3.6 × 10^7^, *P* < 0.0001 *SDHA*; 5.2 × 10^6^ vs. 8.3 × 10^6^, *P* < 0.001 *TBP*; Figure 2B) and stimulated (mean copies/10^6^ cells: 6.9 × 10^8^ vs. 9.3 × 10^8^, *P* < 0.0001 *IFN-*γ; 4.0 × 10^8^ vs. 5.2 × 10^8^ *P* < 0.001 *RPL13a*; 5.1 × 10^6^ vs. 7.3 × 10^6^, *P* < 0.01 *TBP;* Figure 2B) PBMCs. Likewise, there was a significant increase in RNA concentration following RT-qPCR using the MagMAX-RNeasy® MiniElute combination when compared to RNeasy® Mini, for both unstimulated (mean copies/μL_RT:_ 3.8 × 10^6^ vs. 7.2 × 10^6^, *P* < 0.0001 *RPL13a*; 2.3 × 10^5^ vs. 4.2 × 10^5^, *P* < 0.001 *SDHA*; 5.8 × 10^4^ vs. 1.0 × 10^5^, *P* < 0.001 *TBP*; Figure 2B) and stimulated (mean copies/μL_RT:_ 7.7 × 10^6^ vs. 1.4 × 10^7^, *P* < 0.001 *IFN-*γ; 4.5 × 10^6^ vs. 7.7 × 10^6^, *P* < 0.0001 *RPL13a;* 5.7 × 10^4^ vs. 9.2 × 10^4^, *P* < 0.0001 *TBP;* Figure 2B) PBMCs. RNA yield and concentration were not significantly affected by the RNeasy® Micro extraction kit. When assessing technical reproducibility, extraction method also did not significantly affect standard deviation amongst replicate extractions (Supplementary Table 1). These data establish that magnetic bead-based extraction significantly enhanced RT-qPCR signal, as compared to silica column extractions.

### Superscript™ IV Significantly Increased RT-qPCR Gene Expression Signal Measurements

**Figure 3:**
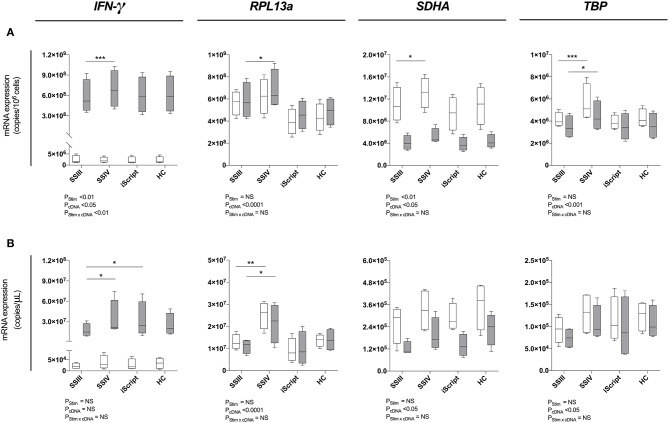
Reverse transcription evaluation. Four reverse transcription kits were evalued for relative qPCR signal for **(A)** maximal RNA yield, or **(B)** maximal RNA concentration. When maximizing RNA yield, RNA was extracted with MagMAX™ *mirVana*™ (MagMAX) Total RNA Isolation Kit (Applied Biosystems); when maximizing concentration, RNA was concentrated with RNeasy® MiniElute Cleanup Kit (QIAGEN). RNA was reverse transcribed with either Superscript™ III (SSIII), Superscript™ IV (SSIV) (both Invitrogen), iScript™ Advanced (iScript) (BioRad) or High-Capacity (HC) (ThermoFisher) reverse transcription kits. RNA was extracted from 1 × 10^6^ PBMCs pared samples, cultured with complete media (white) or PMA/Iono (gray) for 6 h, then *IFN-*γ, *RPL13a, SDHA*, and *TBP* mRNA expression was quantified. Data were compared with a two-way ANOVA (*P ≤ 0.05; **P ≤ 0.01; ***P ≤ 0.001 for *post-hoc* Bonferroni's multiple-comparisons test). Biological replicate (*n* = 4), single RNA extractions, with triplicate reverse transcription reactions per extraction were performed. Sample mean calculated from the mean of the reverse transcription reactions calculated from the mean of the technical triplicate qPCR reactions. Biological mean ± biological SEM are shown.

To identify the optimal reverse transcription kit, Superscript™ III, Superscript™ IV (both ThermoFisher), iScript™ Advanced (Bio-Rad) and High-Capacity (Applied Biosystems) kits were tested in conjunction with MagMAX (Figure 3A) and MagMAX-RNeasy® MiniElute (Figure 3B). A statistically significant enhancement of RT-qPCR signal was observed with Superscript™ IV as compared to Superscript™ III for both unstimulated (mean copies/10^6^ cells: 1.1 × 10^7^ vs. 1.3 × 10^7^ *P* < 0.05 *SDHA;* 4.1 × 10^6^ vs. 5.6 × 10^6^, *P* < 0.001 *TBP*; Figure 3A) and stimulated (mean copies/10^6^ cells: 5.8 × 10^8^ vs. 6.9 × 10^8^, *P* < 0.001 *IFN-*γ*;* 5.9 × 10^8^ vs. 6.8 × 10^8^, *P* < 0.05 *RPL13a;* 3.5 × 10^6^ vs. 4.5 × 10^6^, *P* < 0.05 *TBP;* Figure 3A) PBMCs. Similarly, Superscript™ IV produced the highest RT-qPCR signal following MagMAX-RNeasy® MiniElute extraction-concentration for both unstimulated (mean copies/μL: 1.3 × 10^7^ vs. 2.5 × 10^7^, *P* < 0.05 *RPL13a*; Figure 3B) and stimulated (mean copies/μL: 1.7 × 10^7^ vs. 3.4 × 10^7^, *P* < 0.05 *IFN-*γ; 1.1 × 10^7^ vs. 2.2 × 10^7^, *P* < 0.001 *RPL13a;* Figure 3B*)* PBMCs. When assessing technical reproducibility, extraction had no statistically significant effect on variation between replicate extractions (Supplementary Table 2). These data identify Superscript™ IV as the superior reverse transcriptase kit irrespective of yield or concentration strategy.

### Single Cell Analytical Sensitivity Was Observed Following Magnetic Bead-Based RNA Extraction

**Figure 4:**
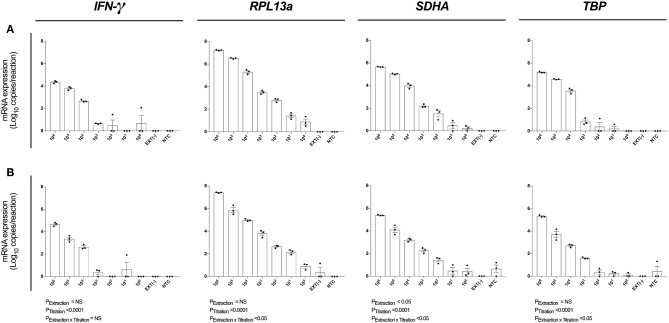
Assay analytical sensitivity. Relative RT-qPCR signal for *IFN-*γ, *RPL13a, SDHA*, and *TBP* mRNA expression from log_10_ dilutions of unstimulated PBMCs when **(A)** maximizing RNA yield, or **(B)** maximizing RNA concentration. When maximizing RNA yield, RNA was extracted with MagMAX™ *mirVana*™ (MagMAX) Total RNA Isolation Kit (Applied Biosystems); when maximizing concentration, RNA was concentrated with RNeasy® MiniElute Cleanup Kit (QIAGEN). All samples were reverse transcribed with Superscript™ IV (Invitrogen). mRNA expression was determined by absolute-quantitative RT-qPCR and gene copy number per reaction was normalized to log_10_ copies per reaction. Biological replicate (*n* = 3), single RNA extractions, with single reverse transcription reactions per extraction were performed. Sample mean calculated from the mean of the technical triplicate qPCR reactions. Biological mean ± biological SEM are shown.

We next evaluated the analytical sensitivity of our optimized protocol using MagMAX extraction kit (Figure 4A) and MagMAX-RNeasy® MiniElute extraction-concentration kit (Figure 4B). RNA was extracted from a log_10_ serial dilution of unstimulated PBMCs and expression of *IFN-*γ*, RPL13a, SDHA*, and *TBP* determined by absolute quantification. The highly expressed *RPL13a* gene was detected at single-cell level from both MagMAX (0.88 log_10_ copies/reaction; Figure 4A) and MagMAX-RNeasy® MiniElute combination (0.90 log_10_ copies/reaction; Figure 4B) extractions. Extraction technique did not influence *IFN-*γ*, RPL13a*, or *TBP* expression; whilst variability in *SDHA* expression (*P*_Ext_ < 0.05) was driven by increased RT-qPCR signal at 10^5^ and 10^4^ PBMCs per extraction. These data establish that our optimized protocol is capable of extracting and quantifying RNA of a highly expressed gene from a single cell. Importantly, the additional step of RNeasy® MiniElute Cleanup did not further enhance analytical sensitivity.

### RT-qPCR Protocol Diagnostic Sensitivity Correlates Significantly With Protein Level Quantification of Epitope-Specific Stimulation From as Few as 1 × 10^4^ PBMCs

**Figure 5:**
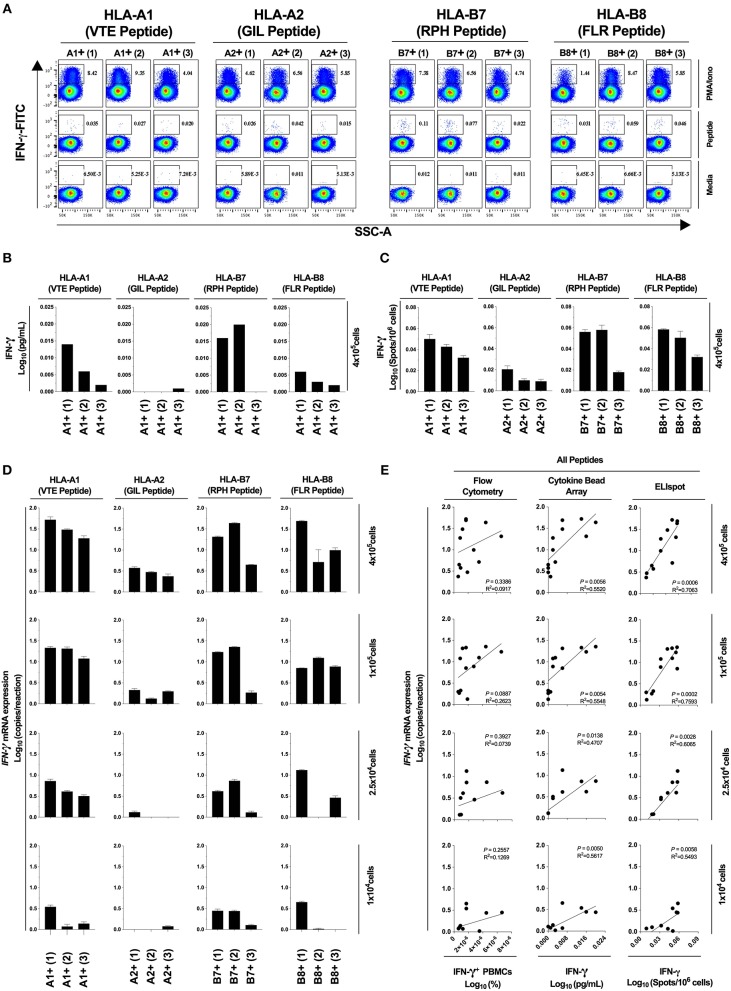
Assay diagnostic sensitivity. Production of IFN-γ protein was determined by flow cytometry **(A)**, bead-based multiplex assay (MagPIX) **(B)**, IFN-γ ELIspot **(C)**; or *IFN-*γ mRNA expression by absolute RT-qPCR **(D)**. *IFN-*γ mRNA expression was correlated to measurements of protein production by flow cytometry, MagPIX or IFN-γ ELIspot (**E**; left, middle and right panel, respectively). Biological replicate (*n* = 3) per HLA-A1, HLA-A2, HLA-B7, or HLA-B8 positive PBMCs (i.e., *n* = 12 total) were stimulated with synthetic HLA-matched peptides representing CMV, Influenza or EBV CD8^+^ T cell epitopes “VTE,” “GIL,” “RPH,” or “FLR” (black), respectively. All samples were cultured with media negative control or PMA/Iono positive control. *IFN-*γ mRNA expression was determined by absolute quantification RT-qPCR of titrated PBMCs (4 × 10^5^, 1 × 10^5^, 2.5 × 10^4^, and 1 × 10^4^); gene copy number per reaction was quantified by standard curve and log_10_ transformed. Single RNA extractions, with single reverse transcription reactions per n, were performed. qPCR performed in technical triplicate replicates. Flow cytometry and MagPIX performed in single wells. ELIspot performed in technical triplicate replicates. Sample mean calculated from the mean of the technical single or triplicates. Biological mean ± technical SEM above background shown.

**Figure 6:**
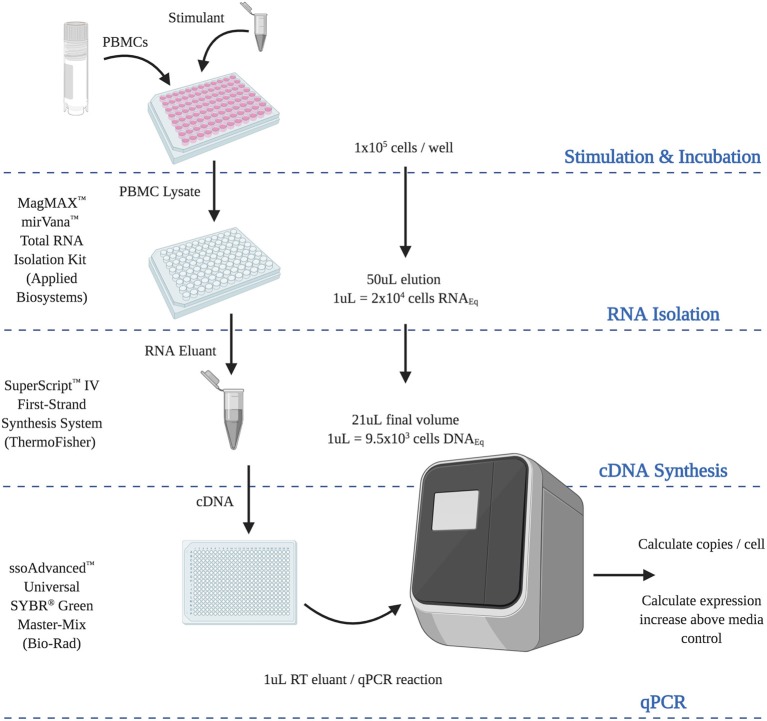
Final optimized RT-qPCR assay. Steps and calculations required for the use of the optimized analytically and diagnostically sensitive RNA extraction and RT-qPCR protocol, from peripheral blood mononuclear cells. (RNA_EQ_, RNA equivalent; DNA_EQ_, DNA equivalent).

Next, we determined the diagnostic sensitivity of our optimized RNA extraction and RT-qPCR protocol to confirm that it accurately reflected data generated using protein-level assays. We evaluated the epitope-specific stimulatory response for four CD8^+^ T cell epitopes restricted by different MHC molecules, quantifying IFN-γ production by flow cytometry, cytokine bead array and ELIspot; and *IFN-*γ mRNA by our optimized protocol. A limited epitope-specific IFN-γ response was demonstrated by flow cytometry (Figure 5A) and bead arrays (Figure 5B) whereas all samples were observed to respond to stimulation by ELIspot (Figure 5C). Importantly, our RT-qPCR protocol (Figure 6), was able to replicate the ELIspot results but with significantly reduced cell numbers (HLA-A1 2.5 × 10^4^, -A2 1 × 10^5^, -B7 1 × 10^4^, -B8 1 × 10^5^; 48-fold, 12-fold, and 48-fold and 12-fold, respectively; Figure 5D).

### Optimized RT-qPCR Assay Correlated With Best-Practice Protein-Based Immunoassays

Next, we assessed correlation between results obtained using our optimized RT-qPCR protocol and current best-practice immunoassays. The correlation between our RT-qPCR protocol and ELIspot was significant at all cell numbers tested [*P* = 0.0006, *R*^2^ = 0.7063 (4 × 10^5^); *P* = 0.0002, *R*^2^ = 0.7593 (1 × 10^5^); *P* = 0.0028, *R*^2^ = 0.6065 (2.5 × 10^4^); *P* = 0.0058, *R*^2^ = 0.5493 (1 × 10^4^); Figure 5E, right panel]. The correlation was also significant with MagPIX multiplex bead-based array [*P* = 0.0056, *R*^2^ = 0.5520 (4 × 10^5^); *P* = 0.0054, *R*^2^ = 0.5548 (1 × 10^5^); *P* = 0.0138, *R*^2^ = 0.4707 (2.5 × 10^4^); *P* = 0.0050, *R*^2^ = 0.5617 (1 × 10^4^); Figure 5E, middle panel], but non-significant (albeit with a similar trend) with flow cytometry (Figure 5E, left panel). Thus, data generated using our optimized RT-qPCR assay are consistent with best practice protein-based immunoassays. Furthermore, our assay is capable of defining an epitope-specific response hierarchy from as few as 1 × 10^4^ cells, representing a clinically and diagnostically meaningful reduction in cell number.

Taken together, we report here a highly sensitive RNA extraction and RT-qPCR quantification strategy using the MagMAX RNA extraction kit, Superscript™ IV reverse-transcription kit and ssoAdvanced™ SYBR master-mix (Supplementary Table 3). This assay is sensitive to the single cell level, can define an epitope hierarchy of response from as few as 1 × 10^4^ cells, and represents a sensitive and robust alternative to protein quantification for research, diagnostic and clinical applications.

## Discussion

Herein, we describe an optimized RNA extraction and RT-qPCR protocol requiring low PBMC numbers, with high analytical and diagnostic sensitivity, whilst maintaining high correlation to protein-level quantification that is typically reliant on much larger cell numbers for detection.

Precise RT-qPCR results are typically dependent on reactions maintaining efficiency close to 100% (45). Both assay design (e.g., primer concentration, master-mix) and sample (e.g., co-extracted inhibitors) may influence PCR efficiency. We made use of the open-access database PrimerBank™ since those primers have been designed for use under consistent conditions (i.e., optimal Tm 60°C) and cover most known human and mouse genes (55). We found primer concentration titrations did not impact reaction efficiency, whereas the SYBR master-mix had a significant impact. PCR inhibitors, including hemoglobin, lactoferrin, anticoagulants, IgG, polysaccharides, and proteases, can be co-extracted in PBMC preparations (56, 57). It is known that some DNA-polymerase variants and PCR buffer “enhancers” have improved reaction efficiency in the presence of such inhibitors (56, 58). The ssoAdvanced™ master-mix, identified herein as optimal of those tested, appears to be one such master-mix facilitating PCR efficiency in the presence of co-extracted inhibitors. Optimization of master-mix reagents will likely continue to be important in improving blood-based PCR analysis and diagnostics (59, 60); especially for accurate amplification of relatively low abundant targets, comparisons between populations with high variability, or amplification from inhibitor-enriched mediums (i.e., whole blood extractions) (10, 59).

We tested three RNA extraction kits by evaluating extraction quality and efficiency: RNeasy® Mini Kit, RNeasy® Micro Kit, and MagMAX™ mirVana™ Kit; in combination with the RNA purification and concentration kit: RNeasy® MiniElute Cleanup Kit. When extracting identically controlled samples, all kits yielded RNA with equivalent RIN scores and low technical variability between replicates. Importantly, RNA yield from PBMCs was significantly increased using MagMAX as compared to silica-column technologies. Additionally, when compared to silica-column extractions, we found MagMAX was more cost and time efficient when running larger number of samples (e.g., 96 samples in ~2 h). We therefore expect magnetic bead-based extractions will become increasingly common within blood-based nucleic acid isolations (59, 61, 62). In addition to extraction techniques (e.g., silica column, phase separation), other factors that could impact RNA quality, yield and concentration include sample collection, storage, and transportation.

Four reverse transcriptase (RT) kits were also evaluated: SuperScript™ III First-Strand Synthesis System, SuperScript™ IV First-Strand Synthesis System, iScript™ Advanced cDNA Synthesis Kit and High-Capacity RNA-cDNA Kit™. Of those, Superscript™ IV was associated with the highest qPCR signal. A previous study evaluating RNA extracted from PBMCs using earlier-generation RT kits reported >128-fold increased qPCR signal between kits (1). We speculate that the reduced variability that we observed between RT kits tested in our study reflects consistent kit quality, purity of the RNA extracted by MagMAX, or a combination thereof.

Both analytical sensitivity and diagnostic sensitivity are key criteria for any RT-qPCR protocol. We show that the analytical sensitivity of our assay is to the level of single cell RNA detection for relatively highly expressed RPL13a. Sample concentration and clean-up has been suggested to remove inhibitors and increase sensitivity (63, 64). Unexpectedly, we found this step did not improve our analytical sensitivity, and was time-consuming, expensive and reduced sample volume. Nevertheless, if concentration is warranted under specific experimental situations, our data suggest that it is technically feasible while retaining high analytical sensitivity. Diagnostic sensitivity determined using MagMAX showed that an epitope response hierarchy could be detected with as few as 1 × 10^4^ PBMCs. It is well-known that there is no absolute correlation between RNA expression and protein translation. Indeed, correlations between transcript and protein expression would be markedly reduced under situations of epigenetic, post-transcriptional or post-translational modification of the gene of interest (65). Nevertheless, we correlated our optimized RT-qPCR assay with commonly used protein-level immunoassays (flow cytometry, cytokine bead arrays, and ELIspot) and showed a very high correlation with the gold-standard protein-level assay, ELIspot, as well as the commonly used MagPIX bead-based cytokine assay, at all tested PBMC concentrations. This highlights that our protocol represents a robust alternative to protein-based assays (e.g., when measuring changes in cytokine mRNA expression in PBMCs in response to specific *in vitro* stimulation). This work will significantly improve analytical capacity of studies relying on irreplaceable, relatively small or costly human samples (e.g., neonatal PBMCs) (66, 67).

Another important outcome of our work is the finding that absolute quantification of transcripts and subsequent normalization to cell numbers is the most appropriate analysis strategy for RNA/RT-qPCR quantification from PBMCs (45, 48). We observed significant alterations in gene expression of commonly used reference genes *RPL13a, SDHA* and *TBP* following stimulation. This is not unexpected as reference genes have been described as variable across cell types, tissues, and experimental and stimulatory conditions (25, 47, 68, 69).

In summary, we report herein the development of an optimized PBMC RNA extraction and RT-qPCR protocol. We employed a qPCR strategy of absolute quantification utilizing PrimerBank™ primers and ssoAdvanced™ Universal SYBR® Green Master-Mix. PBMC RNA was isolated with MagMAX™ *mirVana*™ Total RNA Isolation Kit and reverse transcribed with SuperScript™ IV First-Strand Synthesis System. Our assay provided single cell analytical sensitivity and a diagnostic sensitivity that could define response hierarchy from 1 × 10^4^ cells. This assay offers an alternative to current best practice protein-based immunoassays, especially for limited PBMC numbers. This work has broad applicability for both clinical and primary research practice.

## Data Availability Statement

The raw data supporting the conclusions of this article will be made available by the authors, without undue reservation, to any qualified researcher.

## Ethics Statement

The studies involving human participants were reviewed and approved by James Cook University Human Research Ethics Committee. The patients/participants provided their written informed consent to participate in this study.

## Author Contributions

DB and CL performed experiments. DB, JB, CL, and DD designed experiments. DB, JB, AW, CL, and DD analyzed and interpreted the data. DB, CL, and DD wrote the manuscript, with input from JB and AW.

### Conflict of Interest

The authors declare that the research was conducted in the absence of any commercial or financial relationships that could be construed as a potential conflict of interest.
