# Supplementary material for: An Analytically and Diagnostically Sensitive RNA Extraction and RT-qPCR Protocol for Peripheral Blood Mononuclear Cells
Source: Front Immunol. 2020 Mar 20;11:402. doi: 10.3389/fimmu.2020.00402 (PMC7098950; doi:10.3389/fimmu.2020.00402)
Supplement: Supplementary file 1 [file Data_Sheet_1.PDF]

Supplementary Table 1. Technical and biological variability of RNA extraction kits. The mean standard deviation of technical (kit variability) and biological (sample variability) replicates from RTqPCR analysis of *IFN-γ*, *RPL13a*, *SDHA* and *TBP* expression as determined following RNA extraction kit testing. $1x10^6$ PBMCs were cultured with complete media (Non-Stim) or stimulated (Stim) with PMA/Iono for 6 hours. RNA was extracted using the RNeasy® Mini (Mini) Kit, the RNeasy® Micro (Micro) Kit (both QIAGEN), and the MagMAX™ *mirVana*™ (MagMAX) Total RNA Isolation Kit (Applied Biosystems), with concentration step performed using the RNeasy® MiniElute (+) Cleanup Kit (QIAGEN). All samples were reverse transcribed with Superscript™ III (Invitrogen). Data $\log_{10}$ transformed standard deviation of RNA expression of mean (copies/10^6cells) and (copies/µL). Change in standard deviation (Δ Log10(σ)) calculated as biological variability over technical variability. Data were analyzed using a two-way ANOVA with post-hoc Bonferroni's multiple-comparisons test. * Technical variability greater than biological variability.

Supplementary Table 2. Technical and biological variability of cDNA kits. The mean standard deviation of technical (kit variability) and biological (sample variability) replicates from RTqPCR analysis of *IFN-γ*, *RPL13a*, *SDHA* and *TBP* expression as determined following RNA extraction kit testing. $1x10^6$ PBMCs were cultured with complete media (Non-Stim) or stimulated (Stim) with PMA/Iono for 6 hours. RNA was reverse transcribed with either Superscript™ III (SSIII), Superscript™ IV (SSIV) (both Invitrogen), iScript™ Advanced (iScript) (BioRad) or High-Capacity (HC) (ThermoFisher) reverse transcription kits. Data $\log_{10}$ transformed standard deviation of RNA expression of mean (copies/10^6 cells) and (copies/μL). Change in standard deviation (Δ Log10(σ)) calculated as biological variability over technical variability. Data were analyzed using a two-way ANOVA with post-hoc Bonferroni's multiple-comparisons test.

Supplementary Table 3. Technical guideline for PBMC RNA quantification via RTqPCR. Summary of optimization results for human peripheral blood mononuclear cell (PBMC) RNA extraction and quantification with reverse transcription quantitative PCR (RTqPCR). This assay allows single cell analytical sensitivity and a diagnostic sensitivity that can define immunodominant hierarchy from $1x10^4$ cells.

Supplementary Table 1

	Technical Variability Mean $Log_{10}(\sigma)$		Biological Variability Mean $Log_{10}(\sigma)$		Δ $Log_{10}(\sigma)$	
IFN-γ	Non-Stim	Stim	Non-Stim	Stim	Non-Stim	Stim
Mini	0.210*	0.038	0.182	0.197	-0.027	0.159
Mini (+)	0.235	0.058	0.313	0.183	0.078	0.125
Micro	0.194*	0.053	0.090	0.222	-0.103	0.169
Micro (+)	0.144	0.051	0.228	0.248	0.084	0.196
MagMAX	0.221*	0.069	0.126	0.184	-0.095	0.116
MagMAX (+)	0.264	0.072	0.396	0.230	0.132	0.158
	P_{Ext} = NS					
RPL13a	Non-Stim	Stim	Non-Stim	Stim	Non-Stim	Stim
Mini	0.142	0.024	0.214	0.106	0.072	0.082
Mini (+)	0.045	0.066	0.110	0.116	0.066	0.050
Micro	0.051	0.039	0.120	0.089	0.068	0.051
Micro (+)	0.048	0.038	0.083	0.102	0.035	0.064
MagMAX	0.041	0.074	0.130	0.077	0.089	0.003
MagMAX (+)	0.069	0.064	0.139	0.118	0.070	0.054
	P_{Ext} = NS					
SDAH	Non-Stim	Stim	Non-Stim	Stim	Non-Stim	Stim
Mini	0.180	0.095	0.244	0.103	0.065	0.007
Mini (+)	0.057*	0.292*	0.045	0.099	-0.012	-0.193
Micro	0.100*	0.218*	0.025	0.078	-0.075	-0.140
Micro (+)	0.208*	0.061	0.166	0.111	-0.042	0.051
MagMAX	0.375*	0.102	0.186	0.129	-0.189	0.027
MagMAX (+)	0.150*	0.741*	0.149	0.431	-0.001	-0.310
	P_{Ext} = NS					
TBP	Non-Stim	Stim	Non-Stim	Stim	Non-Stim	Stim
Mini	0.130	0.036	0.206	0.100	0.076	0.063
Mini (+)	0.072	0.089	0.084	0.115	0.013	0.027
Micro	0.079	0.069	0.135	0.129	0.056	0.060
Micro (+)	0.054	0.043	0.067	0.094	0.012	0.051
MagMAX	0.058	0.065	0.225	0.072	0.166	0.006
MagMAX (+)	0.054	0.074	0.084	0.119	0.030	0.045
	P_{Ext} = NS					

Supplementary Table 2

	Technical Variability Mean $Log_{10}(\sigma)$		Biological Variability Mean $Log_{10}(\sigma)$		Δ $Log_{10}(\sigma)$	
IFN-γ	Non-Stim	Stim	Non-Stim	Stim	Non-Stim	Stim
SSIII	0.139	0.055	0.258	0.210	0.119	0.155
SSIV	0.139	0.080	0.260	0.217	0.121	0.137
iScript	0.123	0.092	0.312	0.301	0.189	0.209
HC	0.162	0.065	0.251	0.205	0.088	0.140
	P_{Ext} = NS					
RPL13a	Non-Stim	Stim	Non-Stim	Stim	Non-Stim	Stim
SSIII	0.074	0.065	0.132	0.079	0.057	0.013
SSIV	0.085	0.076	0.167	0.134	0.082	0.058
iScript	0.118	0.115	0.203	0.207	0.085	0.092
HC	0.075	0.082	0.129	0.102	0.055	0.019
	P_{Ext} = NS					
SDAH	Non-Stim	Stim	Non-Stim	Stim	Non-Stim	Stim
SSIII	0.106	0.082	0.118	0.113	0.012	0.031
SSIV	0.081	0.106	0.104	0.106	0.023	0.000
iScript	0.120	0.107	0.189	0.204	0.068	0.097
HC	0.094	0.105	0.122	0.119	0.028	0.014
	P_{Ext} = NS					
TBP	Non-Stim	Stim	Non-Stim	Stim	Non-Stim	Stim
SSIII	0.079	0.065	0.122	0.118	0.044	0.052
SSIV	0.074	0.075	0.140	0.137	0.067	0.062
iScript	0.090	0.088	0.137	0.245	0.046	0.157
HC	0.072	0.079	0.101	0.113	0.029	0.034
	P_{Ext} = NS					

Supplementary Table 3

Workflow Order	Protocol Step		Optimal Kit	Pros	Cons
1	PBMC RNA Extraction		MagMAX™ *mirVana*™ Total RNA Isolation Kit (Applied Biosystems)	- PBMC RNA yield and concentration significantly increased<br>- Low variability between technical replicates<br>- Time and cost efficient<br>- Amendable to automation	- Beads bind total nucleic acid (requires DNase)<br>- Requires specialist equipment (*i.e.* plate shaker and magnetic stand)
2	RNA to cDNA Synthesis		SuperScript™ IV First-Strand Synthesis System (ThermoFisher)	- PBMC RNA yield and concentration significantly increased<br>- Low variability between technical replicates	- Expensive relative to other cDNA synthesis kits
3	qPCR	Reaction Master-mix	ssoAdvanced™ Universal SYBR® Green Master-Mix (Bio-Rad)	- Optimal reaction efficiency and largest dynamic range	- Expensive relative to other SYBR chemistry master-mix kits
		Primer Selection	PrimerBank™	- Minimal primer optimization required<br>- Designed to provide optimal binding at 60°C<br>- Covers most known human and mouse genes	- May not cross introns<br>- May not be as efficient as manually designed primers
		Quantification Strategy	Absolute quantification based on standard curve	- Remove potential reference gene bias post PBMC stimulation	- May introduce technical error if experiment is inappropriately controlled
